# Dichlorotrifluoromethoxyacetic Acid: Preparation and Reactivity †

**DOI:** 10.3390/molecules22060966

**Published:** 2017-06-09

**Authors:** Riadh Zriba, Alaric Desmarchelier, Frédéric Cadoret, Sébastien Bouvet, Anne-Laure Barthelemy, Bruce Pégot, Patrick Diter, Guillaume Dagousset, Jean-Claude Blazejewski, Elsa Anselmi, Yurii Yagupolskii, Emmanuel Magnier

**Affiliations:** 1 Institut Lavoisier de Versailles (ILV), UMR CNRS 8180, Université de Versailles, 45 avenue des Etats-Unis, 78035 Versailles CEDEX, France; riadh.zriba@yahoo.ca (R.Z.); alaric.desmarchelier@gmail.com (A.D.); fredcadoret@yahoo.fr (F.C.); sebastien.bouvet@uvsq.fr (S.B.); anne-laure.barthelemy@uvsq.fr (A.-L.B.); bruce.pegot@uvsq.fr (B.P.); patrick.diter@uvsq.fr (P.D.); guillaume.dagousset@uvsq.fr (G.D.); jcblaz@chimie.uvsq.fr (J.-C.B.); elsa.anselmi@uvsq.fr (E.A.); 2 Infectiologie et Santé Publique (ISP), UMR 1282 INRA/Université de Tours (UFR Sciences & Techniques), Parc de Grandmont, 37200 Tours, France; 3 Institute of Organic Chemistry, National Academy of Sciences of Ukraine, Murmans’ka Str. 5, 02094 Kyiv, Ukraine; yagupolskii@ioch.kiev.ua

**Keywords:** fluorine, trifluoromethoxy, amides

## Abstract

We describe the first gram scale preparation of the reagent dichlorotrifluoromethoxyacetic acid. This stable compound is obtained in five steps starting from the cheap diethylene glycol. The reactivity of the sodium salt of this fluorinated acid was also tested and allowed the preparation of new amides.

## 1. Introduction

Since the seminal preparation of the trifluoromethoxy group by Yagupolskii in 1955 [1], the interest in this very specific organic moiety has grown continuously, in particular for life sciences purposes [2,3,4,5,6,7,8,9]. Such interest can be explained by the conjunction of its multiple advantages: the “pseudohalogen” character of this entity which makes it comparable to a fluorine atom in terms of electronic properties, and the deep modifications of the conformation as well as the physico-chemical behavior induced in molecules linked to this group [10,11,12,13,14]. The unrivaled and promising properties brought by this ether function are in deep contrast with the synthetic difficulties to prepare it [15,16,17]. Major and recent progresses have been made in either the direct introduction of the trifluoromethoxy moiety [18,19,20,21,22,23] (often through a nucleophilic pathway) or in its preparation from alcohols or phenols [24,25,26,27,28]. There is nevertheless still an urgent need for new methods able to selectively introduce this moiety at a late stage of a synthetic procedure. The design of new reagents enabling the grafting of this substituent should be a highly valuable addition to the presently existing methods. Based on our ongoing research project in this field, we thought that related trifluoromethoxy group-bearing molecules should be easily accessible based on previous work of our laboratory [29,30,31,32].

In this communication we describe the preparation of the sodium salt of dichlorotrifluoromethoxyacetic acid, its attempted use in chlorotrifluoromethoxycarbene generation and trapping thereof, as well as the concomitant preparation of some interesting new nitrogen-based trifluoromethoxy-bearing building blocks.

## 2. Results and Discussion

**Scheme 1:**
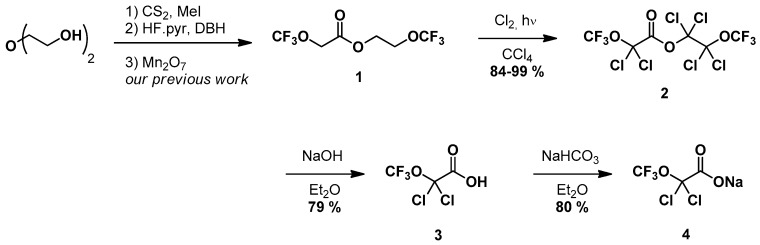
First preparation of dichlorotrifluoromethoxyacetic acid **3**.

The planned synthesis of the target dichlorotrifluoromethoxyacetic acid, **3** (Scheme 1) was based on initial chlorination of the trifluoromethoxy ester **1** previously described by our group [24]. Thus, exhaustive chlorination of ester **1**, dissolved in CCl_4_, under UV irradiation in an intermittent stream of dichlorine easily afforded the perchlorinated ester **2**. ^19^F NMR spectra of this ester exhibited clear evidence for partial restricted rotation as shown by the presence of one sharp (δ = −54.4 ppm) and one very broad peak (δ = −54.5 ppm) instead of the expected two sharp signals. Further saponification of one equivalent of ester **2** with sodium hydroxide gave two equivalents of the unknown targeted acid **3** after acidification of the reaction medium, gratifyingly making use of both trifluoromethoxy groups present in the starting molecule. Free acid **3**, which tenaciously retained diethyl ether solvent, could only be partially characterized and was then transformed in a final step. The derived sodium salt **4** of acid **3** could however be readily isolated in acceptable overall yield from ester **1** after simple treatment of a diethyl ether solution of acid **3** with a stoichiometric amount of sodium bicarbonate followed by thorough drying under high vacuum.

**Scheme 2:**
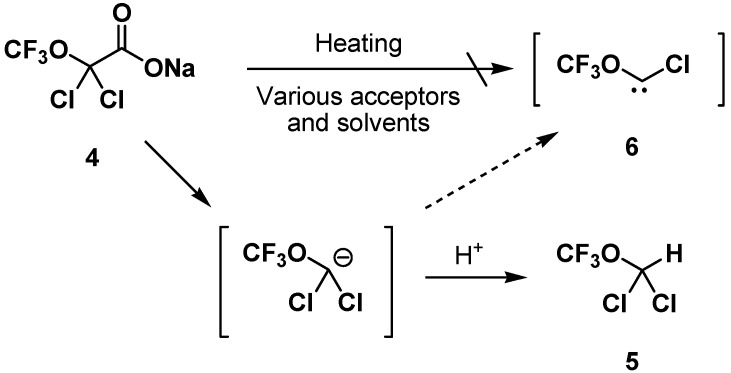
Attempts at carbene generation.

With compound **4** in hand, we then studied the opportunity to generate the chlorotrifluoromethoxycarbene **6** (Scheme 2).

**Scheme 3:**
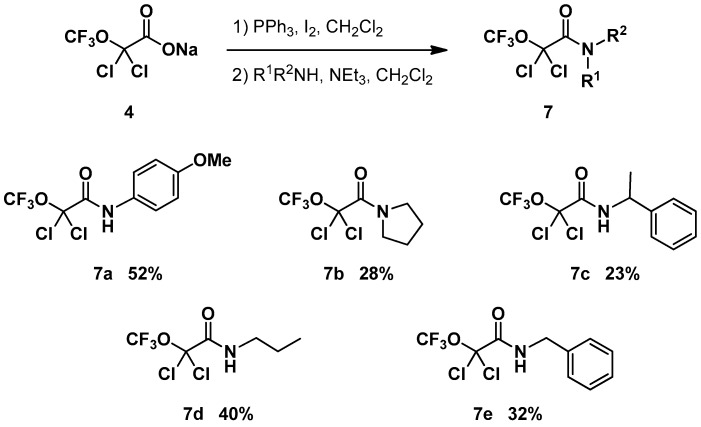
Preparation of various dichlorotrifluoromethoxy acetamides.

Most fluorinated carbenes are known for their electrophilic character. They usually react with electron rich functionalities [33,34,35]. The presence of an oxygen atom adjacent to the carbenic center in the carbenic species **6** (Scheme 2) we planned to generate, was however susceptible to alter this normal behavior [36,37,38]. We thus attempted the trapping of the derived carbene **6** with a wide panel of variously substituted olefins with either electrophilic (trichlorofluoroethene, dimethylbutadiene, etc.) or nucleophilic (enol ethers, etc.) character. Whatever the conditions employed (solvents, temperature, aromatic, double or triple bonds as a trap), no trace of the desired chlorotrifluoromethoxymethylated compounds was obtained. In some cases, the only perfluorinated molecule detected was the dichlorotrifluoromethoxymethane **5** [39]. Even if very volatile, we were able to isolate it by careful distillation (from a crude mixture with diglyme as solvent), yielding small amounts of pure product **5**. The latter was characterized by NMR. The generation of the expected carbene **6** was assumed to proceed in a two-step pathway: first a decarboxylation followed by the elimination of a chlorine atom. The presence of the compound **5** was a formal proof of the success of the first step but also of the inability of the carbanionic intermediate to evolve into a carbenic species. Its final reprotonation (from a proton coming from the reaction medium) delivered then the neutral molecule **5**. In order to assess the shelf stability and utility of salt **4**, we tried to use it in the preparation of some amide derivatives **7**, using an already described method for chlorodifluoroacetic acid (Scheme 3) [40].

As shown by the results depicted in the Scheme 3, these derivatives were isolated in relatively modest but satisfactory yields comparable to those obtained with the sodium salt of chlorodifluoroacetic acid [40]. Aliphatic, benzylic and aromatic amines were suitable for this transformation. Obviously, sodium salt **4** exhibited sufficient stability for normal handling. To the best of our knowledge, none of the compounds **7a**–**e** have been described so far. The reagent **4** is consequently a new building block for the introduction of a trifluoromethoxy moiety.

## 3. Materials and Methods

### 3.1. General Information

Each reaction was carried out under an argon atmosphere in freshly distilled solvent, unless otherwise noted. All chemicals were purchased from commercial sources (Sigma-Aldrich, Saint-Quentin Fallavier, France; ABCR, Karlsruhe, Deutschland or Alfa Aesar, Haverhill, MA, USA) and were used without further purification. Organic solvents were purchased from Merck (Darmstadt, Deutschland) and Carlo Erba (Val-de-Reuil, France). NMR spectra were recorded on AC-200 and AC-300 spectrometers (Bruker, Wissembourg, France). Reported coupling constants and chemicals shifts were based on a first order analysis. Internal reference was the residual peak of CHCl_3_ (7.26 ppm) for ^1^H (200 MHz), central peak of CDCl_3_ (77.1 ppm) for ^13^C (50 MHz) spectra, and internal CFCl_3_ (0 ppm) for ^19^F (188 MHz) NMR spectra. Chemical shifts are reported in parts per million (ppm) and constants *J* in hertz (Hz). Mass spectra (MS) in the positive ion mode (ESI+) were obtained on a Xevo Q-Tof instrument (WATERS, Guyancourt, France). IR spectra were recorded on a Nicolet 400SD spectrophotometer (Thermo Fisher, Villebon-sur-Yvette, France).

*1,1,2,2-Tetrachloro-2-(trifluoromethoxy)ethyl 2,2-dichloro-2-(trifluoromethoxy)acetate* (**2**). Dichlorine gas was bubbled into a solution of ester **1** (3 g, 11.7 mmol) in CCl_4_ (2 mL) contained in a quartz vessel until the solution remained yellow, and was then irradiated with a high-pressure mercury lamp (HPK 125 W Philips, Suresnes, France) for 18–32 h with intermittent bubbling of dichlorine until ^1^H NMR showed complete chlorination (absence of protons). The solvent was evaporated to give essentially pure chlorinated product **2** as a colourless oil (4.5–5.3 g; 84–99% yield). ^19^F NMR (CDCl_3_, 188 MHz): δ = −54.5 (br s), −54.4 (s); ¹³C NMR (CDCl_3_, 50 MHz): δ = 154.0, 120.4 (q, *J* = 267.8 Hz, OCF_3_), 120.2 (q, *J*_CF_ = 268.1 Hz, OCF_3_), 108.9 and 108.7 (2 × br s (rotamers), CCl_2_), 97.2 (q, *J* = 2.5 Hz, 1C, CCl_2_), 87.7 (q, *J* = 1.1 Hz, 1C, CCl_2_); IR (neat): ν = 1757, 1818 cm^−1^.

*2,2-Dichloro-2-(trifluoromethoxy)acetic acid* (**3**). NaOH (163 mg, 4 mmol) solubilized in a minimal amount of water (1.5 mL) was added to a solution of chlorinated ester **2** (940 mg, 2 mmol) in Et_2_O (10 mL). The mixture was vigorously stirred for 6 h at room temperature, acidified with 37% HCl and extracted with diethyl ether (2 × 5 mL), dried over MgSO_4_ and concentrated under vacuum to afford acid **3** still containing diethyl ether. The exact quantity of remaining diethyl ether was quantified by ^19^F and ^1^H NMR using methyl trifluoroacetate as standard (79% corrected yield). ¹H NMR (CDCl_3_, 300 MHz): δ = 8.35 (br s, 1H); ^19^F NMR (CDCl_3_, 188 MHz): δ = −54.3 (s); ¹³C NMR (CDCl_3_, 50 MHz): δ = 163.6, 120.0 (q, *J* = 265.1 Hz, OCF_3_), 98.15 (q, *J* = 2.1 Hz, CCl_2_); MS (ESI+): *m*/*z* = 167.9 [M − CO_2_ + H]^+^; IR (neat): ν = 1629, 1747, 3406, 3467 cm^−1^.

*2,2-Dichloro-2-(trifluoromethoxy)acetic acid sodium salt* (**4**). Powdered sodium bicarbonate (1 equiv) was added in portions to a solution of the preceding acid **3** in diethyl ether (10 mL). The resulting suspension was stirred overnight. The solvent was removed under reduced pressure and the resulting off-white powder was thoroughly dried under high vacuum at room temperature (0.75 g; 80% yield); ^19^F NMR (D_2_O, 188 MHz): δ = −53.8 (s); MS (*m*/*z*): 257.0 (M + Na^+^), 100%; IR (KBr): ν = 1675, 3432 cm^−1^.

*Dichlorotrifluoromethoxymethane* (**5**). ¹H NMR (CDCl_3_, 300 MHz): δ = 7.27 (s, 1H); ^19^F NMR (CDCl_3_, 188 MHz): δ = −60.6 (s); ¹³C NMR (CDCl_3_, 50 MHz): δ = 120.1 (q, *J* = 266.1 Hz, OCF_3_), 90.9 (q, *J* = 5.0 Hz).

*General procedure for the synthesis of amides from sodium 2,2-dichloro-2-(trifluoromethoxy)acetic acid sodium salt* (**4**), *as exemplified by the preparation of 2,2-dichloro-N-(4-methoxyphenyl)-2-(trifluoromethoxy)acetamide* (**7a**). Sodium 2,2-dichloro-2-(trifluoromethoxy)acetate (**4**, 59 mg, 0.25 mmol, 1.0 equiv) was added to a solution of triphenylphosphine (79 mg, 0.30 mmol, 1.2 equiv) and iodine (76 mg, 0.30 mmol, 1.2 equiv) in CH_2_Cl_2_ (3 mL). After 30 min of stirring, a solution of *p*-anisidine (46 mg, 0.38 mmol, 1.5 equiv) and triethylamine (53 µL, 0.38 mmol, 1.5 equiv) in CH_2_Cl_2_ (1 mL) was transferred via cannula in the reaction mixture at room temperature, which was further stirred for 16 h. Water (10 mL) and CH_2_Cl_2_ (5 mL) were then added and the aqueous phase was extracted with CH_2_Cl_2_ (2 × 15 mL). The combined organic layers were dried over magnesium sulfate, filtered and concentrated under vacuum. Purification by silica gel preparative plate (solvent: pentane/Et_2_O 7:3) afforded the expected amide **7a** (41 mg, 52%) as a light brown oil. HRMS calcd. for C_10_H_8_^35^Cl_2_F_3_NNaO_3_: 339.9731; found: 339.9743 (δ = 3.5 ppm). ^1^H NMR (CDCl_3_, 300 MHz): δ = 8.01 (br s, 1H, NH), 7.50–7.45 (m, 2H), 6.93–6.88 (m, 2H), 3.81 (s, 3H); ^19^F NMR (CDCl_3_, 188 MHz): δ = −53.9 (s, 3F, OCF_3_); ^13^C NMR (75 MHz, CDCl_3_): δ = 158.5, 157.8, 128.6, 122.5, 120.3 (q, J = 268.3 Hz, OCF_3_), 114.6, 100.7 (q, J = 2.1 Hz, CCl_2_), 55.6. The following amides were similarly prepared:

*2,2-Dichloro-1-(piperidin-1-yl)-2-(trifluoromethoxy)ethanone* (**7b**). Colorless oil (20 mg, 28%). HRMS calcd. for C_8_H_10_^35^Cl_2_F_3_NNaO_2_: 301.9938; found: 301.9948 (δ = 3.3 ppm). ^1^H NMR (CDCl_3_, 300 MHz): δ = 3.82–3.51 (m, 4H), 1.51–1.78 (m, 6H); ^19^F NMR (CDCl_3_, 188 MHz): δ = −53.9 (s, 3F); ^13^C NMR (CDCl_3_, 75 MHz): δ = 158.2, 120.1 (q, *J* = 267.4 Hz, OCF_3_), 102.8 (q, *J* = 1.8 Hz, CCl_2_), 48.7 and 46.8 (2 × br s (rotamers), 2C), 25.8, 24.3.

*2,2-Dichloro-*N*-propyl-2-(trifluoromethoxy)acetamide* (**7c**). Colorless oil (25 mg, 40%). HRMS calcd. for C_6_H_8_^35^Cl_2_F_3_NNaO_2_: 275.9782; found: 275.9786 (δ = 1.4 ppm). ^1^H NMR (CDCl_3_, 300 MHz): δ = 6.44 (br s, 1H, NH), 3.42–3.25 (m, 2H), 1.73–1.53 (m, 2H), 0.97 (t, *J* = 7.4 Hz, 3H); ^19^F NMR (CDCl_3_, 188 MHz): δ = −54.0 (s, 3F, OCF_3_); ^13^C NMR (CDCl_3_, 75 MHz): δ = 161.1, 120.2 (q, *J* = 267.9 Hz, OCF_3_), 100.6 (q, *J* = 1.8 Hz, CCl_2_), 42.7, 22.5, 11.3.

*N-Benzyl-2,2-dichloro-2-(trifluoromethoxy)acetamide* (**7d**). Colorless oil (24 mg, 32%). HRMS calcd. for C_10_H_8_^35^Cl_2_F_3_NNaO_2_: 323.9782; found: 323.9781 (δ = −0.3 ppm). ^1^H NMR (CDCl_3_, 300 MHz): δ = 7.43–7.28 (m, 5H), 6.74 (br s, 1H, NH), 4.55 (d, *J* = 5.8 Hz, 2H); ^19^F NMR (CDCl_3_, 188 MHz): δ = −54.0 (s, 3F, OCF_3_); ^13^C NMR (CDCl_3_, 75 MHz): δ = 161.1, 136.4, 129.2, 128.4, 128.0, 120.2 (q, *J* = 268.1 Hz, OCF_3_), 100.2 (q, *J* = 2.0 Hz, CCl_2_), 45.0.

*(R)-2,2-Dichloro-N-(1-phenylethyl)-2-(trifluoromethoxy)acetamide* (**7e**). Colorless oil (18 mg, 23%). HRMS calcd. for C_11_H_10_^35^Cl_2_F_3_NNaO_2_: 337.9938; found: 337.9951 (d = 3.8 ppm). ^1^H NMR (CDCl_3_, 300 MHz): δ = 7.44–7.27 (m, 5H), 6.56 (br s, 1H, NH), 5.11 (quint, *J* = 7.1 Hz, 1H), 1.60 (d, *J* = 6.9 Hz, 3H); ^19^F NMR (CDCl_3_, 188 MHz): δ = −53.9 (s, 3F, OCF_3_); ^13^C NMR (CDCl_3_, 75 MHz): δ = 160.1, 141.3, 129.1, 128.2, 126.3, 120.2 (q, *J* = 268.1 Hz, OCF_3_), 100.6 (q, *J* = 1.9 Hz, 1C, CCl_2_), 50.7, 21.2.

## 4. Conclusions

An easy access to the sodium salt **4** of dichlorotrifluoromethoxyacetic acid was devised. Attempted trapping of chlorotrifluoromethoxycarbene generated by decarboxylation of this salt with alkenes failed presumably because of the poor reactivity of this carbene under the conditions used for its formation. Nevertheless, salt **4** proved sufficiently stable for the preparation of new trifluoromethoxylated-bearing amide synthons **7a**–**e**. Improved precursors of trifluoromethoxycarbene are under current development in our laboratories. We are studying in particular the preparation of chlorofluorotrifluoromethoxyacetic acid.

## References

[1] Yagupolskii L.M. (1956). Synthesis of derivatives of phenyl trifluoromethyl ethers. *Dokl. Acad. Nauk SSSR* 1955, *105*, 100–102. Chem. Abstr..

[2] Zeng Z., Gao T., Li Y., Wang X., Yang X., Wu M. (2016). Synthesis and biological activity of arylsulfonamide derivatives containing 2-arylamino-4(3*H*)-quinazolinone. J. Pest. Sci..

[3] Xu D., Guan J., Xu X., Gong S., Xu H. (2016). A New Method for the Synthesis of Oxadiazine Insecticide Indoxacarb. J. Heterocycl. Chem..

[4] Liu A., Wang X., Liu X., Li J., Chen H., Hu L., Yu W., He L., Liu W., Huang M. (2017). Synthesis and Fungicidal Activity of Novel 2-Heteroatomthiazole-based Carboxanilides. J. Heterocycl. Chem..

[5] Alverez C., Arkin M.R., Bulfer S.L., Colombo R., Kovaliov M., LaPorte M.G., Lim C., Liang M., Moore W.J., Neitz R.J. (2015). Structure−Activity Study of Bioisosteric Trifluoromethyl and Pentafluorosulfanyl Indole Inhibitors of the AAA ATPase p97. ACS Med. Chem. Lett..

[6] Wu C., Sun X., Feng C., Liu X., Wang H., Feng F., Zhang J. (2016). Proton-Coupled Organic Cation Antiporter Contributes to the Hepatic Uptake of Matrine. J. Pharm. Sci..

[7] Yamamoto S., Ohta H., Abe K., Kambe D., Tsukiyama N., Kawakita Y., Moriya M., Yasuhara A. (2016). Identification of 1-Methyl-*N*-(propan-2-yl)-*N*-({2-[4-(trifluoromethoxy)-phenyl]pyridin-4-yl}methyl)-1*H*-imidazole-4-carboxamide as a Potent and Orally Available Glycine Transporter 1 Inhibitor. Chem. Pharm. Bull..

[8] Satam V.S., Pedada S.R., Kamraj P., Antao N., Singh A., Hindupur R.M., Pati H.N., Thompson A.M., Launay D., Martin D. (2017). Development of a Scalable Process for the Synthesis of DNDI-VL-2098: A Potential Preclinical Drug Candidate for the Treatment of Visceral Leishmaniasis. Org. Proc. Res. Dev..

[9] Kang S., Kim Y.M., Kim R.Y., Seo M.J., No Z., Nam K., Kim S., Kim J. (2017). Synthesis and structure-activity studies of side chain analogues of the anti-tubercular agent, Q203. Eur. J. Med. Chem..

[10] Aldrich P.E., Sheppard W. (1964). α-Fluorinated Ethers. II. Alkyl Fluoroalkyl Ethers. J. Org. Chem..

[11] Haas A. (1984). The Element Displacement Principle: A New Guide in P-Block Element Chemistry. Adv. Inorg. Chem..

[12] Olah G.A., Yamamoto T., Hashimoto T., Shih J.G., Trivedi N., Singh B.P., Piteau M., Olah J.A. (1987). Aromatic substitution. 53. Electrophilic nitration, halogenation, acylation, and alkylation of α,α,α-trifluoromethoxybenzene. J. Am. Chem. Soc..

[13] Kondratov I.S., Logvinenko I.G., Tolmachova N.A., Morev R.N., Kliachyna M.A., Clausen F., Daniliuc C.G., Haufe G. (2017). Synthesis and physical chemical properties of 2-amino-4-(trifluoromethoxy)butanoic acid –CF_3_O-containing analogue of natural lipophilic amino acids. Org. Biomol. Chem..

[14] Feng P., Lee K.N., Lee J.W., Zhen C., Ngai M.-Y. (2016). Access to a New Class of Synthetic Building Blocks via Trifluoromethoxylation of Pyridines and Pyrimidines. Chem. Sci..

[15] Leroux F., Manteau B., Vors J.-P., Pazenok S. (2008). Trifluoromethyl ethers-synthesis and properties of an unusual substituent. Beilstein J. Org. Chem..

[16] Besset T., Jubault P., Pannecoucke X., Poisson T. (2016). New entries toward the synthesis of OCF_3_-containing molecules. Org. Chem. Front..

[17] Tlili A., Toulgouat F., Billard T. (2016). Synthetic Approaches to Trifluoromethoxy-Substituted Compounds. Angew. Chem. Int. Ed..

[18] Kolomeitsev A.A., Vorobyev M., Gillandt A. (2008). Versatile application of trifluoromethyl triflate. Tetrahedron Lett..

[19] Marrec O., Billard T., Vors J.-P., Pazenok S., Langlois B. (2010). A New and Direct Trifluoromethoxylation of Aliphatic Substrates with 2,4-Dinitro(trifluoromethoxy)benzene. Adv. Synth. Catal..

[20] Huang C., Liang T., Harada S., Lee E., Ritter T. (2011). Silver-Mediated Trifluoromethoxylation of Aryl Stannanes and Arylboronic Acids. J. Am. Chem. Soc..

[21] Sokolenko T.M., Davydova Y.A., Yagupolskii Y.L. (2012). Efficient synthesis of 5′-fluoroalkoxythiazoles via α-bromo-α-fluoroalkoxyacetophenones Hantzsch type cyclization with thioureas or thioamides. J. Fluor. Chem..

[22] Chen C., Chen P., Liu G. (2015). Palladium-Catalyzed Intramolecular Aminotrifluoromethoxylation of Alkenes. J. Am. Chem. Soc..

[23] Guo S., Cong F., Guo S., Wang L., Tang P. (2017). Asymmetric silver-catalysed intermolecular bromotrifluoromethoxylation of alkenes with a new trifluoromethoxylation reagent. Nat. Chem..

[24] Fantasia S., Welch J.M., Togni A. (2010). Reactivity of a Hypervalent Iodine Trifluoromethylating Reagent toward THF: Ring Opening and Formation of Trifluoromethyl Ethers. J. Org. Chem..

[25] Liu J.-B., Chen C., Chu L., Chen Z.-H., Xu X.-H., Qing F.-L. (2015). Silver-Mediated Oxidative Trifluoromethylation of Phenols: Direct Synthesis of Aryl Trifluoromethyl Ethers. Angew. Chem. Int. Ed..

[26] Liu J.-B., Xu X.-H., Qing F.-L. (2015). Silver-Mediated Oxidative Trifluoromethylation of Alcohols to Alkyl Trifluoromethyl Ethers. Org. Lett..

[27] Liang A., Han S., Liu Z., Wang L., Li J., Zou D., Wu Y., Wu Y. (2016). Regioselective Synthesis of *N*-Heteroaromatic Trifluoromethoxy Compounds by Direct O−CF_3_ Bond Formation. Chem. Eur. J..

[28] Zhou M., Ni C., He Z., Hu J. (2016). *O*-Trifluoromethylation of Phenols Access to Aryl Trifluoromethyl Ethers by *O*-Carboxydifluoromethylation and Decarboxylative Fluorination. Org. Lett..

[29] Blazejewski J.-C., Anselmi E., Wakselman C. (2001). 2-Trifluoromethoxyethyl Triflate: A Versatile Trifluoromethoxyethyl Carrier. J. Org. Chem..

[30] Blazejewski J.-C., Anselmi E., Wernicke A., Wakselman C. (2002). Synthesis of 2-trifluoromethoxyethyl trifluoromethoxyacetate and derived 2-trifluoromethoxyacrylates. J. Fluorine Chem..

[31] Magnier E., Diter P., Blazejewski J.-C. (2008). The trifluoromethoxy group as a fluorine twin in the Diels-Alder reactions of halogenated quinones. Tetrahedron Lett..

[32] Zriba R., Magnier E., Blazejewski J.-C. (2009). Preparation and reactivity of some new keto and styrene based trifluoromethoxylated synthons. Synlett.

[33] Brahms D.L.S., Dailey W.P. (1996). Fluorinated Carbenes. Chem. Rev..

[34] Moss R.A., Zhang M., Krogh-Jespersen K. (2009). A New Synthesis of Difluorodiazirine and the Absolute Reactivity of Difluorocarbene. J. Am. Chem. Soc..

[35] Moss R.A. (2010). “Carbon Dichloride”: Dihalocarbenes Sixty Years after Hine. J. Org. Chem..

[36] Moss R.A., Zhang M. (2008). Activation Parameters for Additions of Ambiphilic Methoxychlorocarbene to Alkenes. Org. Lett..

[37] Moss R.A., Zhang M., Krogh-Jespersen K. (2010). The Nucleophilic Reactivity of Fluoromethoxycarbene. Org. Lett..

[38] Grygorenko O.O., Artamonov O.S., Komarov I.V., Mykhailiuk P.K. (2011). Trifluoromethyl-substituted cyclopropanes. Tetrahedron.

[39] Scherer O., Millauer H. (1968). Fluoro ethers.

[40] Bordeau M., Frébault F., Gobet M., Picard J.-P. (2006). Ethyl Difluoro(trimethylsilyl)acetate and Difluoro(trimethylsilyl)acetamides-Precursors of 3,3-Difluoroazetidinones. Eur. J. Org. Chem..

